# Performance analysis of hyperbolic graded topological resonator for biosensing applications

**DOI:** 10.1038/s41598-025-94062-6

**Published:** 2025-03-17

**Authors:** Diptimayee Dash, Jasmine Saini, Amit Kumar Goyal

**Affiliations:** 1 Department of Electrical and Electronics Engineering, Galgotias College of Engineering and Technology, Greater Noida, 201310 India; 2 Department of Electronics and Communication Engineering, Jaypee Institute of Information Technology, Noida, 201310 India; 3 Department of Electronics and Communication Engineering, Manipal Institute of Technology, Manipal Academy of Higher Education, Manipal, 576104 India

**Keywords:** Graded index, Photonic crystal, Biosensor, Topological resonator, Optical sensors, Microresonators, Photonic crystals, Nanophotonics and plasmonics

## Abstract

In this manuscript, a novel hyperbolic-graded nano-photonic resonator structure based on the photonic crystal is proposed for biosensing applications. The graded refractive index is realized by considering a porous silicon material having a deliberately modulated local refractive index. The introduction of grading effectively modifies its dispersion characteristics. These crystals exhibit overlapping bandgaps and opposite Zak phases, enabling the manifestation of unique topological properties. The design parameters are optimized to excite a topological edge state at a 1521 nm operating wavelength, whereas a resonating TES is excited at a 1533 nm. The structure performance is analyzed using the finite element method. The analytical results exhibit an improved sensitivity of 1806 nm/RIU (refractive index unit) and a Figure of Merit (FOM) of 4030 RIU^−1^, which are 151% and 2483% higher than recently reported values. With its remarkable performance metrics, the proposed device holds significant promise for accurately detecting and sensing biochemical samples with very high efficiency.

## Introduction

The development of efficient biosensors for early cancer detection has been a focus for researchers due to the increasing global incidence of cancer^1^. Recent medical technology and research advancements have significantly improved our ability to detect and diagnose cancer early, leading to better treatment outcomes. Traditional methods for detecting cancerous cells in blood samples, such as microscopic examination, immunophenotyping, and genetic analysis, have limitations in terms of sensitivity, specificity, and manual analysis time. Innovative approaches utilizing nanophotonic techniques and computational methods have emerged in cancer detection, enhancing the accuracy and efficiency of identifying cancerous cells. Photonic crystals (PhCs) have gained significant interest in this area. PhCs are periodic structures with a photonic band gap (PBG) in their transmittance/reflection spectrum, making them suitable for various applications, including cancer detection. The tunable PBG characteristics of 1D-PhC structures is widely explored to design tunable multi-channel filters, sensors and reflectors^2–5^. These devices are further explored to develop tunable phase-controlled devices, biosensors, photonic-spin hall effects devices, thermal sensors, and noise eliminators^6–8^.

The device performance is enhanced by modifying the conventional PhC’s structure. This is accomplished by introducing a defect layer, which breaks the periodicity and alters the dispersion characteristic of the device. This leads to the excitation of resonant modes within the PBG. The excited resonant modes are highly sensitive to a small surrounding perturbation, making defected PhCs valuable for sensing applications. However, the device performance can further be enhanced by considering the thickness or refractive index gradient profile. These graded photonic crystal (1D-GPhC) structures consist of stacked layers with gradual refractive index and thickness modifications. These modifications result in significant dispersion properties, enabling tunable photonic bandgap (PBG) engineering and group velocity control. Gaufillet et al.^9^ demonstrated the impact of the filling factor in the normal direction to control electromagnetic waves in 1D-GPhC structures. Similarly, the impact of graded-index materials on PBG is explored by Singh et al.^10^. Most of the existing researches on 1D-GPhCs are focused on exploring exponential, linear, and sawtooth type grading in various materials^11–18^. However, there has been limited investigation and development of 1D-GPhC structures incorporating hyperbolic graded index materials. The hyperbolic variations of refractive indices in PhC structures hold great potential for integrating optical devices, offering novel and intriguing effects. The gradual change in refractive index, combines the benefits of other graded configurations. This provides precise control over mode confinement, significantly reduced interface losses and dispersion management. The rapid advancement of various 1D-GPhC structures has opened up numerous possibilities for tunable photonic devices such as optical reflectors, sensors, and filters^19–21^.

The device performance and light-matter interaction can further be improved by considering the topological aspects of the structure. The topological aspects offer the excitation of topologically protected edge states (TES). The excited TES shows their robustness against surrounding perturbations and exhibits propagation of low scattered edge modes. Recently, 1D-PhCs have also been utilized to excite TES for sensing applications^22–24^. A TES-based refractive index sensor utilizing 1D-PhC having a sensitivity of 254.5 nm/RIU is proposed by Qing et al.^25^. The sensitivity is enhanced to a 616 nm/RIU value by incorporating an electro-optical (EO) material between the two 1D-PhC structures^26^. The TES concept in 1D-PhC is further extended to study the plasmonic properties of the device. Lu et al.^27^ investigated the enhancement of plasmonic Tamm states using the topological property of a periodic structure. Further, a graphene-based multichannel absorber is proposed by coupling the topological states with Tamm plasmon polaritons^28^. The authors reported a more than 97% absorption in the incidence angle range of 0°–50°. Gao et al. used three 1D-PhC structures and a defect layer to excite a Fano resonance using the structure’s combined topological property and Fabry-Perot cavity^29^. Recently in 2022, TES in 1D-PhC along with three-dimensional Dirac semimetal is utilized to realize a low threshold optical bistability^30^. Furthermore, a polarization-independent optical biosensor has also been proposed to have a sensitivity of $70^\circ$/RIU ($60^\circ$/RIU) for TE (TM) polarized light^31^.

**Fig. 1:**
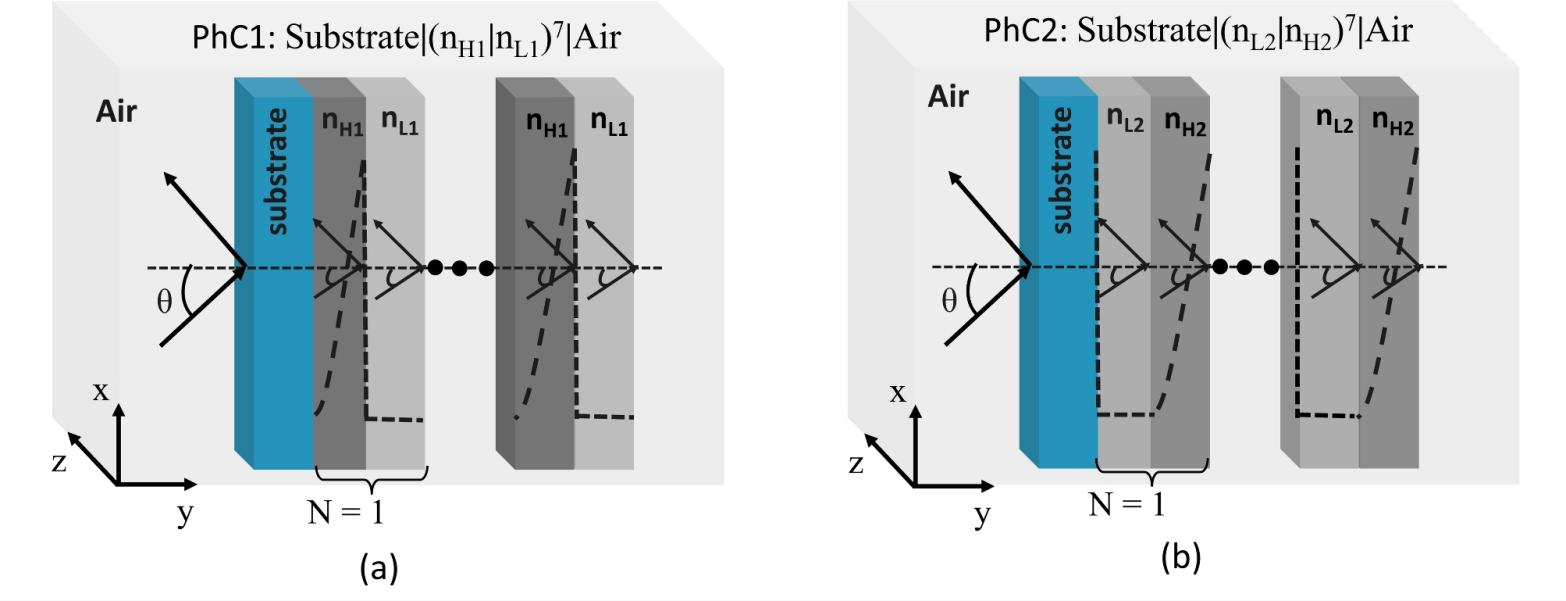
Schematical representation of proposed (**a**) PhC1 structure “Substrate | ($n_{\text {H1}}$, $n_{\text {L1}})^7$ | Air”, and (**b**) PhC2 structure “Substrate | ($n_{\text {L2}}$, $n_{\text {H2}})^7$ | Air”.

Therefore, 1D-PhC structures possess the capability to have superior topological properties, which can be used to manipulate light-matter interactions and thus shows their potential applications in low-concentration analyte detection with the improved figure of merit (FOM). However, to the best of our knowledge, the topological behavior of hyperbolic graded PhC (HGPhC) is still not explored. The HGPhC technique incorporates topological analysis, hyperbolic graded geometry, and PhC structures, which assign specific attributes to different structure components. This approach is expected to have extensive potential applications in biosensing, aiding in early detection for timely intervention and improved patient outcomes. It may also enhance the accuracy of cancer staging, providing valuable information for treatment planning and disease monitoring. Further research and validation studies are needed to fully explore the capabilities of these techniques and integrate them into clinical practice.

This paper analyzes the localization of topological interface modes for a hyperbolic-graded 1D-PhC structure. The TES is excited by optimizing Two hyperbolic graded 1D-PhC structures of overlapped PBG made of silicon and porous silicon materials. The analytical results are verified using the finite element method (FEM) of COMSOL Multiphysics. The parameters are optimized to regulate and alter the dispersion characteristics, which results in the opposite topological properties (Zak Phase) at the overlapping bandgap region. This exhibits the excitation of TES for 1521 nm operating wavelength at the interface having very high electric field intensity. The topological cavity is formed by replacing the two interface layers with an aqueous defect layer. The impact of varying defect layer thickness on excited topological cavity interface mode is also studied in detail. Infiltrating the defect layer with different cancer cell analytes (such as Jurkat, Hela, PHC12, MDA MB-231, and MCF-7) leads to a redshift in the topological cavity resonance wavelength, which is measured to calculate the device’s sensitivity. The analytical results exhibit an average sensitivity of 1806 nm/RIU, having a quality factor and FOM of 4.371 $\times$ $10^{3}$ and 4.03 $\times$ $10^{3}$ RIU^−1^, respectively. The proposed structure shows 151% higher sensitivity, 2483% improvement in the FOM, and 2011% higher quality factor^32^. Finally, the device performance characteristics are compared with the recently data available in literature. This shows the superior sensing performance of the device for medical and commercial applications. Additionally, the structure can easily be fabricated using anodic etching method.

## Theoretical analysis and methods

**Fig. 2:**
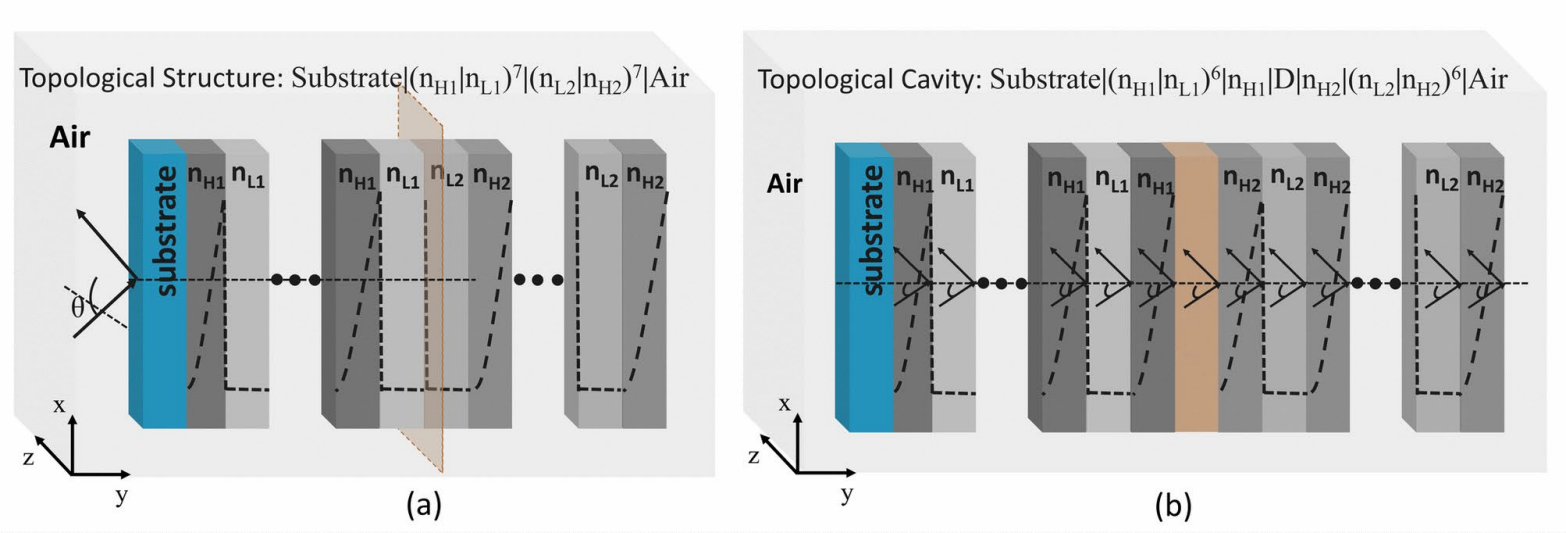
Schematical representation of proposed (**a**) Topological structure “Substrate | ($n_{\text {H1}}$, $n_{\text {L1}})^7$ | ($n_{\text {L2}}$, $n_{\text {H2}})^7$ | Air”, and (**b**) Topological resonator “Substrate | ($n_{\text {H1}}$, $n_{\text {L1}})^6$ | $n_{\text {H1}}$, | D,| $n_{\text {H2}}$,| ($n_{\text {L2}}$, $n_{\text {H2}})^6$ | Air”.

The schematic representation of the proposed hyperbolic graded 1D-PhC heterostructure is shown in Fig. 1. The structures are made of silicon material, where porosity (Eq. 2) is introduced to obtain the graded index profile. Silicon’s wavelength-dependent refractive index (RI) is determined using the Sellmeier approximation^33,34^. The proposed GPhC structure consists of two types of dielectric layers. The high index layer exhibits a hyperbolic variation in refractive index along the thickness, while the low index layer is homogeneous with a constant refractive index. Figure 1a demonstrates the configuration of the PhC1 structure, referred to as [Substrate | ($n_{\text {H1}},$  $n_{\text {L1}})^7$ | Air], having a step-index low RI ($n_{\text {L1}}$) of 1.6 (80% porosity) and a hyperbolic varying high index ($n_{\text {H1}}$) layer from 1.6 (80% porosity) to 3.45 (0% porosity). This gives a PBG1 of 1237–1864 nm for considered layer thicknesses $d_{\text {L1}}$ and $d_{\text {H1}}$ of 242 nm and 153 nm, respectively. The thickness values are calculated considering the quarter wave Bragg stack having a central operating wavelength of 1550 nm. Similarly, PhC2 structure of configuration [Substrate | ($n_{\text {L2}}$, $n_{\text {H2}})^7$ | Air] is shown in Fig. 1b. It is also a GPhC configuration composed of two types of dielectric layers, a step-index low RI ($n_{\text {L2}}$) of 1.8 (70% porosity) and a hyperbolic varying high index ($n_{\text {H2}}$) layer from 1.8 (70% porosity) to 3.45 (0% porosity). This gives a PBG2 of 1270–1826 nm for the considered layer thicknesses $d_{\text {L2}}$ and $d_{\text {H2}}$ of 215 nm and 148 nm, respectively. Introducing porosity makes it possible to achieve the desired graded structure physically. The combined structure to excite TES is shown in Fig. (2).

1 $$\begin{aligned} n_H^2-1=\frac{10.6684293\lambda ^2}{\lambda ^2-0.301516485^2}+\frac{0.0030434748\lambda ^2}{\lambda ^2- 1.13475115^2}+\frac{1.54133408\lambda ^2}{\lambda ^2-1104^2} \end{aligned}$$

The graded index profile can be obtained by introducing the porosity within the material. This also enables effective infiltration of analytes. A layer’s porosity (P) can be analytically calculated using Eq. (2)^35^.

2 $$\begin{aligned} P=\frac{(n_p^2-n_{d s}^2)(n_a^2-2n_{d s}^2)}{(n_p^2+2n_{d s}^2)(n_a^2-n_{d s}^2)}. \end{aligned}$$

In Eq. (2), the refractive indices $n_{\text {p}}$, $n_{\text {a}}$, and $n_{\text {ds}}$ represent the refractive indices of the porous material, air/analytes, and dense material, respectively. The effective RI of high-index material in PhC1 is considered the average RI (1.6–3.45), while the effective RI of high-index material in PhC2 is considered the average RI (1.8–3.45). The hyperbolic graded RI from the initial position d(0) to the end of the layer thickness d(y) is computed using the Eq. (3)

3 $$\begin{aligned} n_{\text {H}}(y) = \frac{n_{\text {i}}}{(1-\alpha y)}. \end{aligned}$$

**Fig. 3:**
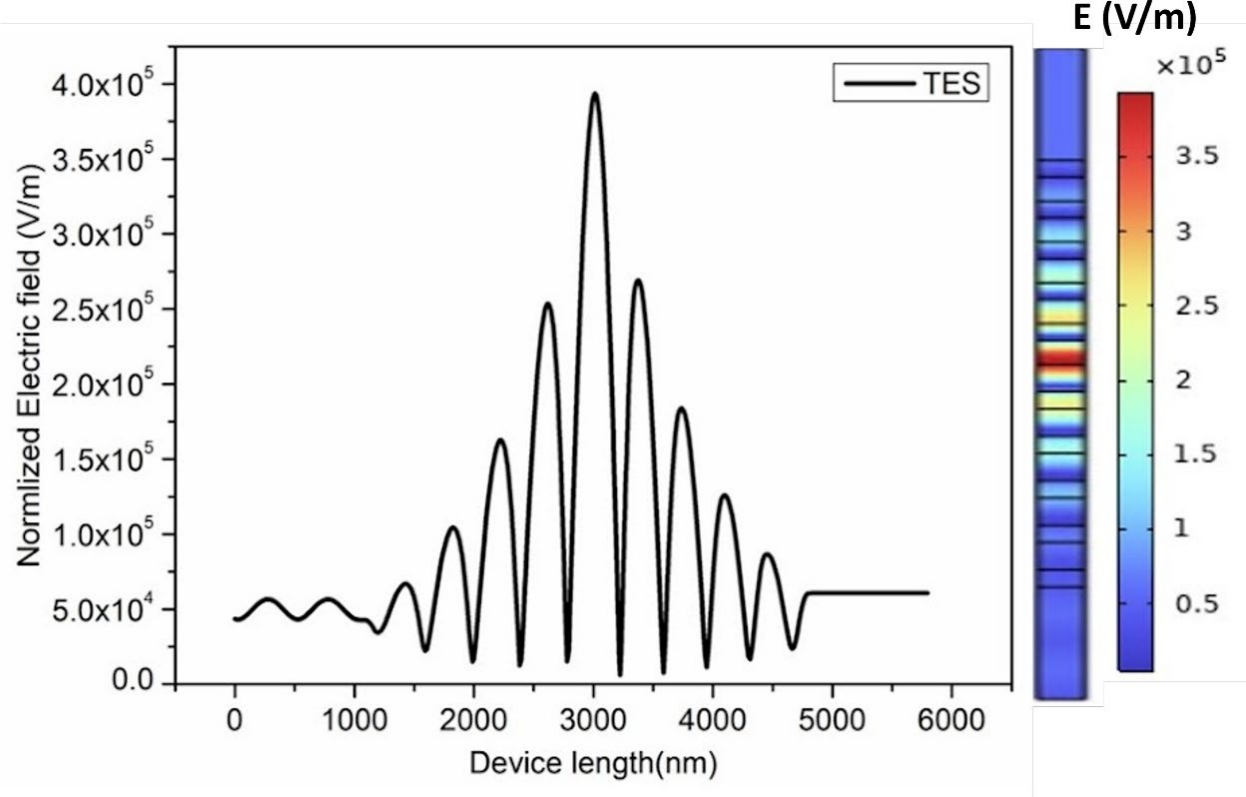
Electrical field distribution within the hyperbolic graded topological structure for TES wavelength of 1521 nm.

Here, $\alpha$ represents the hyperbolic grading parameter, evaluated using the Eq. (4).

4 $$\begin{aligned} \alpha = \frac{n_{\text {f}}-n_{\text {i}}}{n_{\text {f}}\times d}. \end{aligned}$$

The initial and final refractive indices of the layer with a hyperbolic gradient are represented by $n_{\text {i}}$ and $n_{\text {f}}$, respectively. In this context, ‘i’ corresponds to the initial position, ‘f’ denotes the final point along the layer’s thickness, and ‘d’ represents the layer’s thickness. The electric field distribution along the plane perpendicular to the hyperbolic grading layer’s surface is calculated using Eqs. (5)–(7) and is shown in Fig. 3^10,16,35^.

5 $$\begin{aligned} & \textrm{E}_{\textrm{H}}(\textrm{y})= \sqrt{\beta _H} (A_H \cos \left( m \log \left( \beta _H\right) \right) +B_H \sin \left( m \log \left( \beta _H\right) \right) ). \end{aligned}$$

6 $$\begin{aligned} & \beta _{\text {H}} = 1-\alpha \times x. \end{aligned}$$

7 $$\begin{aligned} & m^{2} = \left( \frac{\omega n_i}{C\alpha }\right) ^2\,- 0.25. \end{aligned}$$

where $A_{\text {H}}$ and $B_{\text {H}}$ are hyperbolic graded index layer constants. At a normal incidence angle, the propagation wave vector is presented by the variable $\beta _{\text {H}}$, having $\omega$ as the angular frequency and ‘c’ as the velocity of light. The structure shows a TES excitation having a maximum normalized electric field confinement of about 3.9$\times 10^{5}$ V/m.

The topological states can be excited at the interface of two 1D-PhC structures having overlapping bandgaps and different topological properties or Zak phase. Figure 2a depicts the proposed graded index structure (“Substrate | PhC1 | PhC2 | Air”) to excite a topological edge state, which comprises two one-dimensional photonic crystals (“Substrate | ($n_{\text {H1}}$, $n_{\text {L1}})^7$ | ($n_{\text {L2}}$, $n_{\text {H2}})^7$ | Air”). Further, a topological resonator structure is designed considering a defective cavity layer made by combining two middle-low index layers. Figure 2b shows the topological resonator structure denoted as “Substrate | ($n_{\text {H1}}$, $n_{\text {L1}})^6$ | $n_{\text {H1}}$,| D,| $n_{\text {H2}}$,| ($n_{\text {L2}}$, $n_{\text {H2}})^6$ | Air”. In this resonator, the defect layer thickness ($t_{\text {dd}}$) equals the sum of $d_{\text {L1}}$ and $d_{\text {L2}}$.

**Fig. 4:**
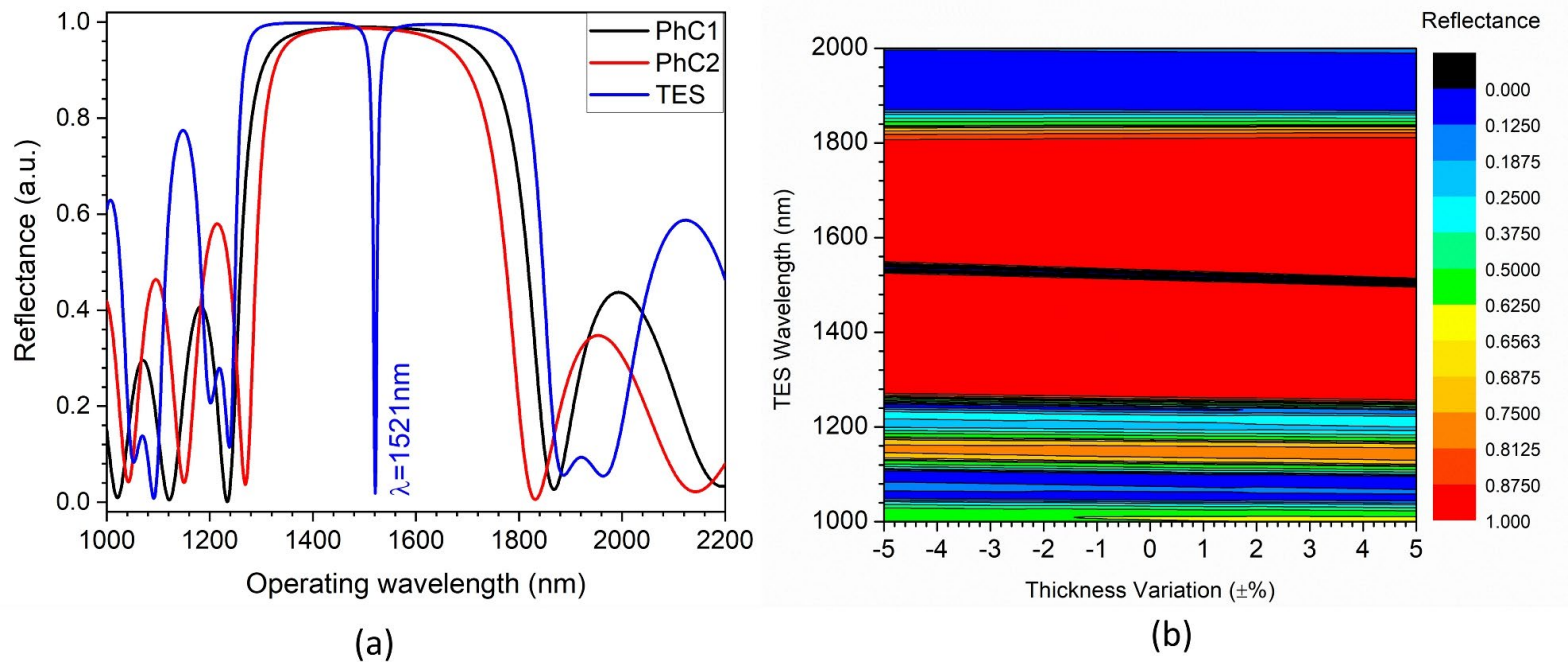
Reflectance response of (**a**) PhC1, PhC2 and combined hyperbolic graded topological structure, and (**b**) Impact of structural thickness variation on exited TES.

## Results and discussion

The structural performance evaluation involves the application of finite element method (FEM). Excitation of topological states can occur at the interface of two 1D-PHC structures with overlapping bandgaps and distinct topological properties or Zak phase. As evident from Fig. 4, the reflectance response of PhC1 and PhC2 exhibits a PBG of width 627 nm (1237–1864 nm) and 556 nm (1270–1826 nm), respectively. The optimization of structural parameters ensures the presence of overlapping bandgaps (1270–1826 nm) in the considered structure. Furthermore, the opposite nature of the Zak phase enhances the likelihood of exciting a topological state at the interface of the two PHC structures. The Zak phase generally has quantized phase values of 0 or $\pi$ and can be calculated from the eigenfrequency band of 1D-PhC as described in Eq. (8)^36^.

**Fig. 5:**
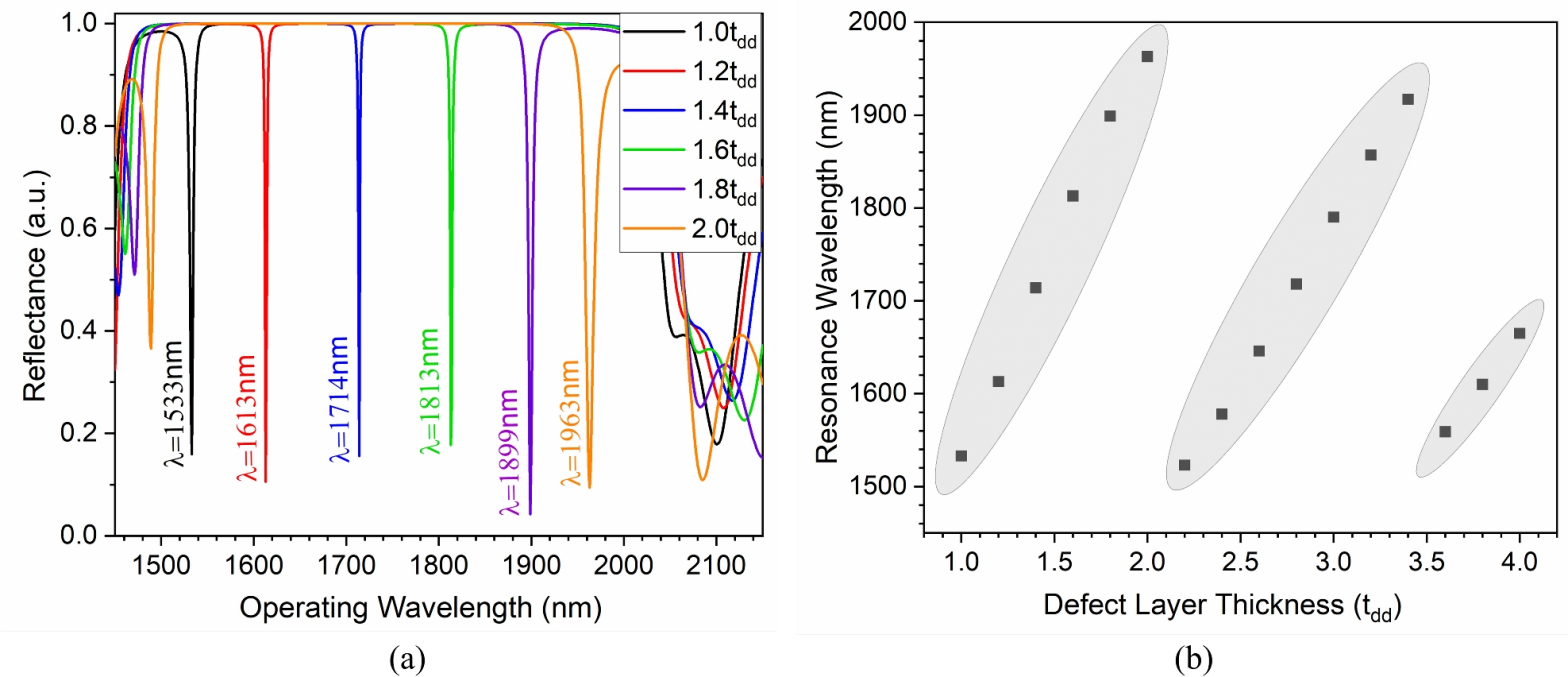
Impact of defect layer thickness on (**a**) reflectance response and (**b**) resonance wavelength of HG-topological resonator structure at normal incidence.

**Fig. 6:**
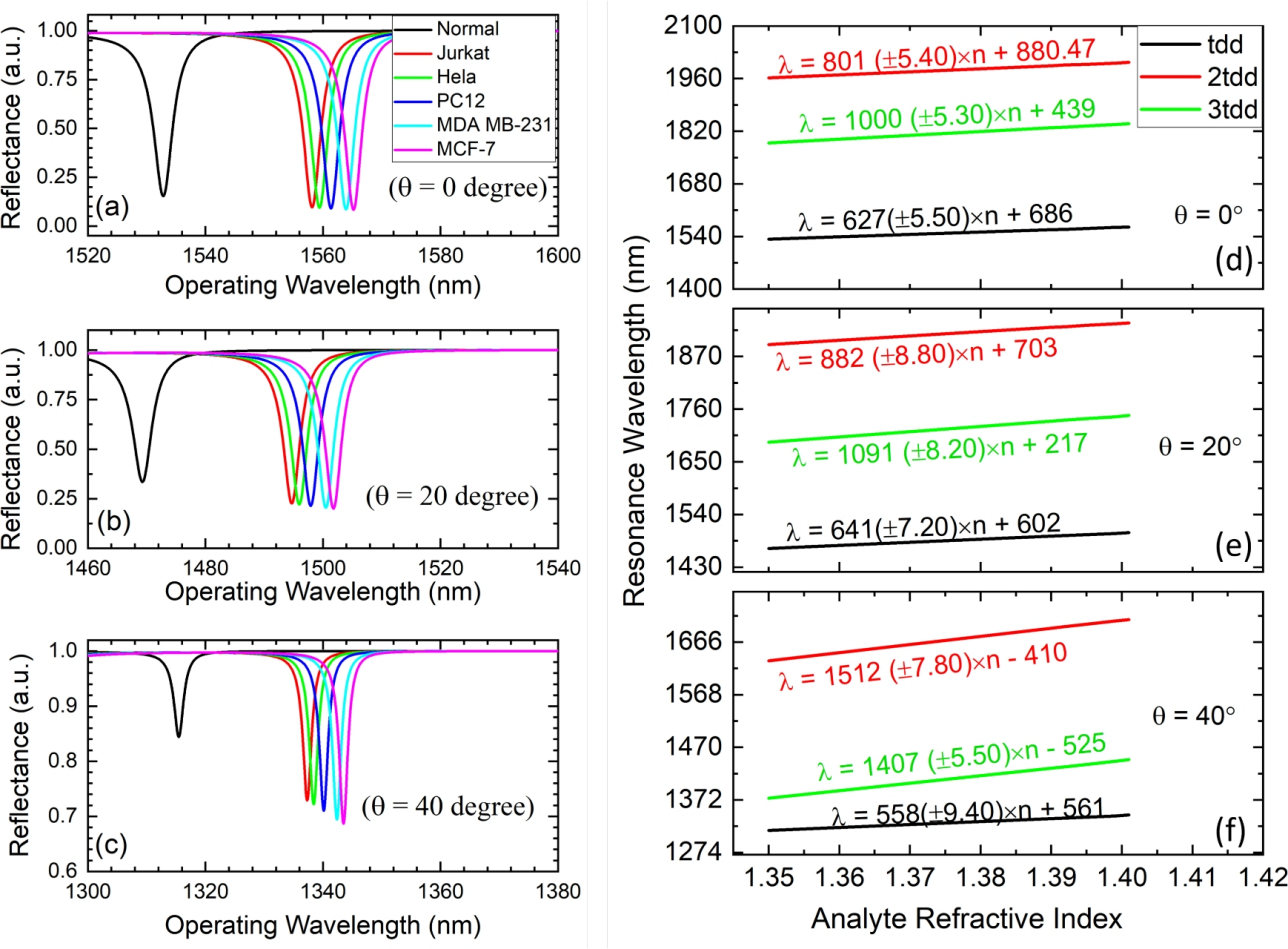
Reflectance response of structure Topological resonator “Substrate | ($n_{\text {H1}}$, $n_{\text {L1}})^6$ | $n_{\text {H1}}$,| D,| $n_{\text {H2}}$,| ($n_{\text {L2}}$, $n_{\text {H2}})^6$ | Air” for defect layer thickness of $t_{dd}$ at varying incidence angle (**a**) 0-degree, (**b**) 20-degree, (**c**) 40-degree, and (**d**–**f**) Comparative impact analysis of infiltrated analyte on resonance wavelength at various incidence angles and defect layer thicknesses.

8 $$\begin{aligned} \theta _n^{Z a k}=\int _{-\pi / \Lambda }^{\pi / \Lambda }\left[ i \int _{\text{ unit } \text{ cell } } d z \varepsilon (z) u_{n, k}^*(z) \partial _k u_{n, k}(z)\right] d k \end{aligned}$$

where k is the wave vector, $\epsilon (z)$ is the dielectric function, $u_{(n,k)}$(z) is the Bloch electric field eigenfunction of the $n_{th}$ band. Thereby, calculating the Zak phase for individual PBG provides topological properties. Thus, the structure shown in Fig. 2a is anticipated to induce a topological state at the interface of two PhCs. This structure exhibits a TES excitation at 1521 nm specifically for the hyperbolic graded topological configuration, as shown in Fig. 4a. Additionally, the excited TES shows robustness against structural variation. As represented in Fig. 4b, the proposed structure exhibits around 1% change in TES wavelength with a corresponding thickness change of 10% (±5%). The excited TES in this structure displays a characteristic Lorentzian curve shape. Furthermore, the topological cavity structure shows the excitation of the topological resonance state at a 1533 nm operating wavelength.

**Table 1:** Cancerous cell refractive index^37^.

Cancer cell type	Refractive index
Normal	1.350
Jurkat	1.390
Hela	1.392
PHC12	1.395
MDA MB-231	1.399
MCF-7	1.401

Although the excited TES is robust, the topological resonator’s excited resonance mode is highly dependent on defect layer thickness. Thus, the analysis is extended to see the impact of defect layer thickness on the excitation characteristics of the TES resonance mode, which is shown in Fig. 5. Increasing the defect layer thickness from 1.0$t_{dd}$ to 2.0$t_{dd}$ leads to the excitation of lower energy resonating TES. The structure shows resonating TES excitation at 1533 nm, 1613 nm, 1714 nm, 1813 nm, 1899 nm, and 1963 nm operating wavelengths for corresponding defect layer thicknesses of 1.0$t_{dd}$, 1.2$t_{dd}$, 1.4$t_{dd}$, 1.6$t_{dd}$, 1.8$t_{dd}$, and 2.0$t_{dd}$, respectively, as shown in Fig. 5a. Additionally, the proposed structure shows a very narrow FWHM of less than 0.5 nm, thus showing its potential in tunable sensing applications. It is noteworthy to mention that increasing the defect layer thickness further leads to again excitation of higher energy resonating TES having almost similar operating wavelength variations ($\frac{d\lambda }{d t_{dd}}$), as shown in Fig. 5b. This provides an added advantage of increased defect layer thickness to enhance light-matter interaction, hence sensitivity.

**Table 2:** Sensitivity of the HG- topological sensor at different incidence angles and defect layer thickness.

Defect layer thickness!	Analyte RI	Sensitivity (nm/RIU)		
		0-degree	20-degree	40-degree
tdd	Jurkat	625	650	550
	Hela	629	643	547
	PHC12	622	644	555
	MDA MB-231	632	633	551
	MCF-7	627	647	568
2tdd	Jurkat	800	900	1525
	Hela	809	880	1523
	PHC12	800	888	1511
	MDA MB-231	795	877	1510
	MCF-7	803	882	1509
3tdd	Jurkat	1000	1100	1400
	Hela	976	1095	1404
	PHC12	977	1088	1400
	MDA MB-231	979	1102	1408
	MCF-7	980	1098	1411
4tdd	Jurkat	1000	1075	1825
	Hela	1000	1071	1809
	PHC12	1000	1088	1800
	MDA MB-231	1000	1081	1795
	MCF-7	1000	1078	1803

### Sensing analysis

The device sensor capability is investigated by considering different cancerous cell analytes. The impact of varying the concentration of infiltrating analytes along with the different defect layer thicknesses on resonating wavelengths is investigated. Introducing porosity facilitates the infiltration of analytes into the defect layer, causing a change in effective RI. This, in turn, leads to a shift in the wavelength of resonating TES mode. Different cancer cells are used as a defect layer analyte, and their refractive indices are tabulated in Table 1^37^. The sensitivity (S), detection limit (DL), sensor resolution (SR), figure of merit (FOM), and signal-to-noise ratio (SNR) of the sensor play a critical role. It can be evaluated using Eqs. (9)–(13)^17,38^.

9 $$\begin{aligned} S= & \frac{\Delta \lambda }{\Delta n}. \end{aligned}$$

10 $$\begin{aligned} FOM= & \frac{S}{FWHM}. \end{aligned}$$

11 $$\begin{aligned} SNR= & \frac{\Delta \lambda _r}{FWHM}. \end{aligned}$$

12 $$\begin{aligned} SR= & \frac{FWHM}{1.5 \times (SNR)^{0.25}}. \end{aligned}$$

13 $$\begin{aligned} DL= & \frac{SR}{S}. \end{aligned}$$

where ‘S’ represents the sensitivity determined by the change in the resonant peak position ($\Delta \lambda$) relative to the resonant peak of the normal cell ($\lambda _{cancerous \, cell} - \lambda _{normal \, cell}$), and $\Delta$n denotes the difference in refractive indices between the sensing cancerous cell and the normal cell ($n_{cancerous \, cell} - n_{normal \, cell}$). The figure of merit (FOM) is determined by the ratio of sensitivity to FWHM and is calculated using Eq. (10), where FWHM represents the spectral half-width of the resonant wavelength dip. The signal-to-noise ratio (SNR) is obtained using Eq. (11), with $\Delta \lambda _r$ being the shift in the resonant wavelength. The sensor resolution (SR) is the smallest spectral shift observable and can be calculated using Eq. (12). Finally, detection limit (DL) is calculated using Eq. (13), which represents the minimum refractive index change detectable by the sensor and is determined by the ratio of the sensor’s resolution (SR) to sensitivity^38,39^.

**Fig. 7:**
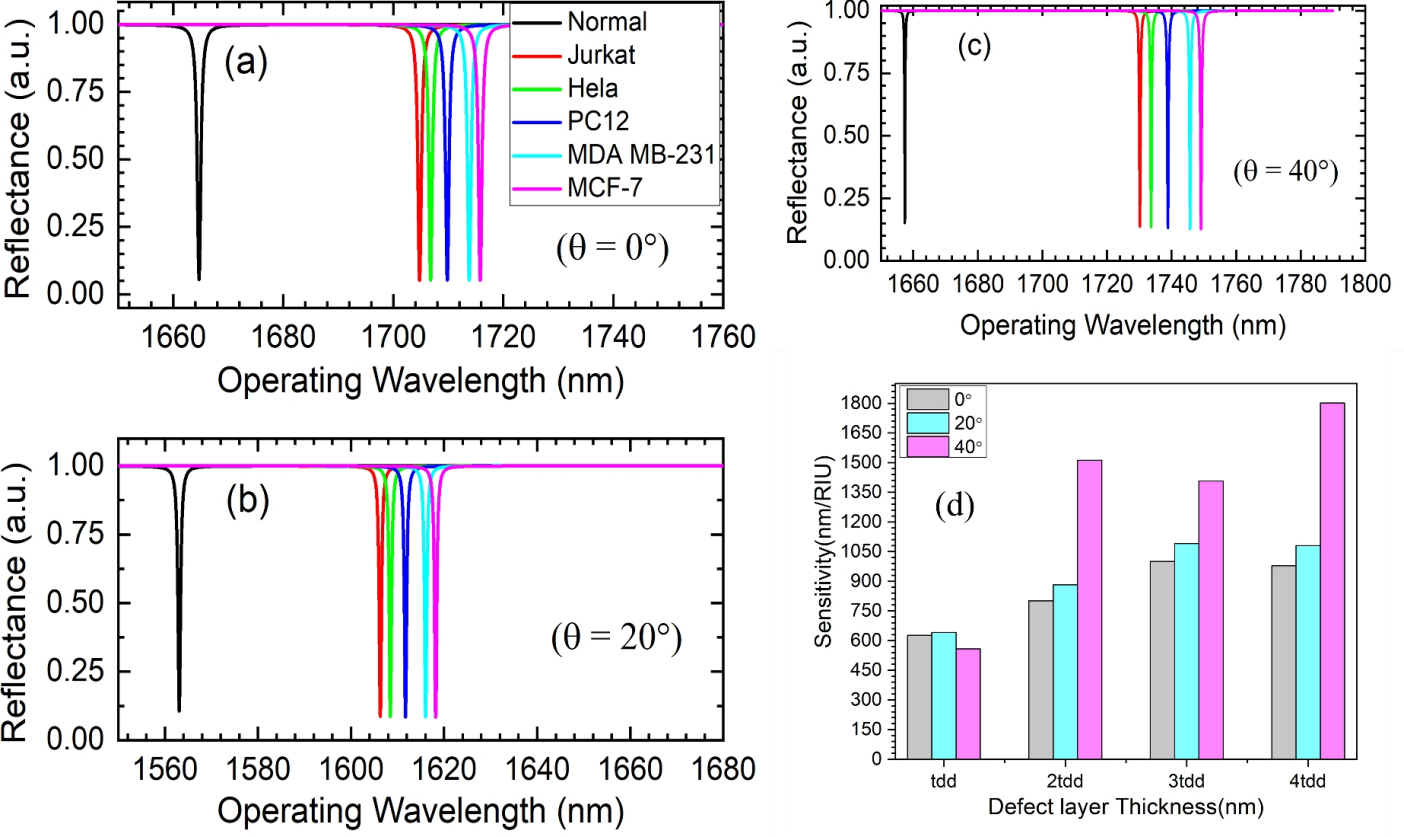
Reflectance response of structure Topological resonator “Substrate | ($n_{\text {H1}}$, $n_{\text {L1}})^6$ | $n_{\text {H1}}$,| D,| $n_{\text {H2}}$,| ($n_{\text {L2}}$, $n_{\text {H2}})^6$ | Air” for defect layer thickness of 4.0$t_{dd}$ at varying incidence angle of (**a**) 0-degree, (**b**) 20-degree, (**c**) 40-degree, and (**d**) Combined sensitivity comparison.

The impact of analyte infiltration of resonating TES at different incidence angles is analytically evaluated. The study indicates that optimizing defect layer thickness at various incidence angles can enhance the sensor’s sensing performance. The device sensing performance is compared at different angles of incidence (0-degrees, 20-degrees, and 40-degrees) for a varying defect layer thickness from 1.0$t_{dd}$ to 4.0$t_{dd}$. Figure 6a–c illustrates the reflectance response of the proposed structure at 1.0$t_{dd}$ defect layer thickness having different infiltrated analytes (cancerous cell), including normal cell (Zero cancerous cell having RI 1.350), jurkat (RI 1.390), hela (RI 1.392), PHC12 (RI 1.395), MDA MB-231 (RI 1.399), and MCF-7 (RI 1.401) at the varying incidence angles of 0-degree, 20-degree and 40-degrees. The analysis is extended for defect layer thicknesses of 2.0$t_{dd}$, 3.0$t_{dd}$, and 4.0$t_{dd}$, respectively. The corresponding analysis is shown in Fig. 6d–f. The infiltration of the analyte leads to an increase in the effective index of the defect layer, which results in a redshift of resonance TES wavelength. Moreover, the sensitivity is also increased for higher defect layer thickness for a given incidence angle. This is because of the increased light-matter interaction. The infiltrated analyte refractive index (n) dependent shift in resonance wavelength for different defect layer thicknesses can be calculated by Eqs. (14)–(16). This gives an average sensitivity of around 600 nm/RIU, 801 nm/RIU, and 1000 nm/RIU for the 1.0$t_{dd}$, 2.0$t_{dd}$, and 3.0$t_{dd}$ defect layer thickness at a 0-degree incidence angle. The average sensitivity increases to 641 nm/RIU, 882 nm/RIU, and 1091 nm/RIU for the 1.0$t_{dd}$, 2.0$t_{dd}$, and 3.0$t_{dd}$ defect layer thickness at a 20-degree incidence angle.

14 $$\begin{aligned} & \lambda _{t_{d d}}^0=627( \pm 5.50) \times n+686 \end{aligned}$$

15 $$\begin{aligned} & \lambda _{2.0 t_{d d}}^0=801( \pm 5.40) \times n+880.47 \end{aligned}$$

16 $$\begin{aligned} & \lambda _{3.0 t_{d d}}^0=1000( \pm 5.30) \times n+439 \end{aligned}$$

The comparative results and structural performance parameters have been summarized in Table 2 for the HGPhC resonator structure. The Table 2 illustrates the correlation between sensitivity, defect layer thickness, and incidence angle, revealing an increase in sensitivity as the thickness expands from 1.0$t_{dd}$ to 4.0$t_{dd}$ at different incident angles of 0-degree, 20-degree, and 40-degrees, respectively. The sensitivity response of the proposed hyperbolic graded topological cavity structure for 4.0$t_{dd}$ defect layer thickness at varying incidence angles is shown in Fig. 7a–c. The 4.0$t_{dd}$ defect layer thickness shows very narrow reflectance peaks having average FWHM of 0.41 nm, where resonating TES is excited at 1657 nm, 1730 nm, 1733 nm, 1738 nm, 1745 nm and 1749 nm for corresponding cancer cell infiltration. This gives an average high-quality factor of around 4.371 $\times$ $10^{3}$, and a maximum average sensitivity of about 1806 nm/RIU, which gives the average FOM of about 4030 RIU^−1^.

The obtained average sensitivity, FOM, and quality factor values are 151%, 2483%, and 2011% better than recently reported values^32^. Therefore, the proposed structure exhibits its potential application as a refractive index-based biosensor. The sensing performance of the hyperbolic graded topological cavity structure for 4.0$t_{dd}$ defect layer thickness at 40-degree incidence angles is summarized in Table 3, and a comparative analysis is presented in Fig. 7d. Finally, the structural performance has been compared with the recently reported values in Table 4. It is evident from Table 4 that the proposed biosensor outperforms current bio-sensing research efforts in terms of average sensitivity, FOM, and quality factor, exhibiting substantially higher performance^40–46^.

**Table 3:** Sensing performance summary of the topological resonator at 4$t_{dd}$ width and 40-degree incidence angle.

Cavity width	Cancer cell	Resonance wavelength (nm)	FWHM (nm)	Quality factor	Sensitivity (nm/RIU)	FOM (RIU^−1^)
1828 nm	Normal	1657	0.24208	6844.845	–	–
	Jurkat	1730	0.40992	4220.336	1825	4452.088212
	Hela	1733	0.42447	4082.738	1809.524	4263.019766
	PHC12	1738	0.45947	3782.619	1800	3917.557185
	MDA MB-231	1745	0.46326	3766.783	1795.918	3876.695592
	MCF-7	1749	0.49514	3532.334	1803.922	3643.256453

**Table 4:** Comparative performance analysis with recently reported results.

References	Average sensitivity (nm/RIU)	FOM (RIU^−1^)	Quality factor	Year
Skin Vitiligo^43^	1200	–	4.065 $\times$ $10^{4}$	2025
Cholesterol^44^	410	247	910	2024
Liver cancer cell^45^	1033	–	721	2023
Cancer cells^46^	406.84	1765.53	535.44	2023
Cancerous blood cells^47^	623	73950	1.2956 $\times$ $10^{5}$	2021
Detection of blood plasma and cancer cells^19^	71.25	–	21.59	2021
Proposed work	1806.8728	4030.5234	4.371 $\times$ $10^{3}$	2025

## Conclusion

A novel bio-photonic resonator design aimed at detecting cancerous blood cells has been proposed in this paper. The design utilizes a topological hyperbolic graded structure, which capitalizes on the unique properties arising from the connection between two photonic crystals having overlapping bandgaps and opposite Zak phases. By incorporating a hyperbolic graded refractive index profile, the dispersion characteristics of the device are effectively modified. The presence of a defect layer within the structure leads to a distinct and prominent resonant peak within the photonic bandgap. The reflectance spectrum is analyzed using the Finite Element Method. Remarkably, the proposed graded topological structure exhibits a sensitivity of 1806 nm/RIU and an impressive FOM of 4030 RIU^−1^. Overall, the proposed device holds great promise for highly efficient and accurate detection and sensing of cancerous blood samples.

## Data Availability

The data underlying the results presented in this paper may be obtained from the corresponding author (A.K.G) upon reasonable request.
